# Supplementary material for: Diagnostic performance of a Recombinant Polymerase Amplification Test—Lateral Flow (RPA-LF) for cutaneous leishmaniasis in an endemic setting of Colombia
Source: PLoS Negl Trop Dis. 2021 Apr 28;15(4):e0009291. doi: 10.1371/journal.pntd.0009291 (PMC8081229; doi:10.1371/journal.pntd.0009291)
Supplement: S5 Table — (DOC) [file pntd.0009291.s007.doc]

**S5 Table. Sensitivity of RPA-LF in the reference laboratory scenario stratified by sociodemographic and clinical characteristics**

| **Characteristic** | **Positive results** | **Sensitivity** | |
| --- | --- | --- | --- |
|  | **RPA-LF/composite gold standard** | **%** | **(95% CI)** |
| **Sociodemographic** |  |  |  |
| Sex |  |  |  |
| Male | 52/60 | 86.7 75.4% 94.1% | (75.4 - 94.1) |
| Female | 20/23 | 87.0 66.4% 97.2% | (66.4 - 97.2) |
| Ethnicity |  |  |  |
| Afro-Colombian | 42/52 | 80.8 | (67.5 - 90.4) |
| Indigenous | 12/13 | 92.3 | (64.0 - 99.8) |
| Mestizo/ Mulatto | 18/18 | 100 | (81.5 - 100) |
| Age (years) |  |  |  |
| < 12 | 9/9 | 100 | (64.4 - 100) |
| ≥ 12 | 63/74 | 85.1 | (75.0 - 92.3) |
| **Clinic** |  |  |  |
| Previous episode of leishmaniasis |  |  |  |
| Yes | 2/2 | 100 | (15.8 -100) |
| No | 70/81 | 86.4 | (77.0 - 93.0) |
| Received any medication last month |  |  |  |
| Yes | 36/44 | 81.8 | (67.3 - 91.8) |
| No | 36/39 | 92.3 | (79.1 - 98.4) |
| Number of lesions |  |  |  |
| 1 - 3 | 58/66 | 87.9 | (77.5 - 94.6) |
| 4 - 11 | 14/17 | 82.4 | (56.6 - 96.2) |
| Duration of the oldest lesion, (months) |  |  |  |
| < 6 | 70/80 | 87.5 | (78.2 - 93.8) |
| > 6 | 2/3 | 66.7 | ( 9.4 - 99.2) |
