# Supplementary material for: Diagnostic performance of a Recombinant Polymerase Amplification Test—Lateral Flow (RPA-LF) for cutaneous leishmaniasis in an endemic setting of Colombia
Source: PLoS Negl Trop Dis. 2021 Apr 28;15(4):e0009291. doi: 10.1371/journal.pntd.0009291 (PMC8081229; doi:10.1371/journal.pntd.0009291)
Supplement: Attachment — Submitted filename: Response to reviwers.docx [file pntd.0009291.s008.docx]

**ANSWERS TO REVIEWERS**

# METHODS

- **Reviewer #1: (No Response)**
- **Reviewer #2:**

1) Line 277: please include primer sequences in the M&M section

We included the reference to the article of our group where the primers and probe are detailed (Saldarriaga et al. 2016). Additionally, the following sentence was added to the final version of the manuscript, lines 281-282.

*“In this study we utilized the primer sequences and probe described by Saldarriaga et al.”*

2) Line 315 calculation methods (formulas) for sensitivity, specificity and positive  / negative predictive should be added, taken that it is not always clear which golden standard is used exactly in the calculation.

Calculations methods were the same as those regularly used in diagnostic test evaluations. The composite gold standard was described in lines 152-164 and 221-228 (initial version of manuscript), and lines 164 – 167 (final version).

Sensitivity, specificity, positive and negative predictive values were estimated using the software Stata®, version 12 and DTCompair. They can be calculated manually using the formulas described by Gordis et al (1). Definitions of true positives and negatives are described in the manuscript (Table 1). Additionally, the raw values of true positive (TP) and negative (TN) values and, false positive (FP) and negative (FN) values are shown in table 3. They allow to confirm manually the estimations performed in the study.

In order to clarify the observations made by the reviewer, we add the reference number 34 (Line 321), which describes the formulas in detail, helping readers to understand how they can be calculated. Additionally, we included this new sentence in the final version, lines 167 -169.

*“The same composite gold standard was used to evaluate the performance of RPA-LF test in both previously described scenarios”.*

- **Reviewer #3:**

1) Lines 148-150: The authors could clarify for which hypothesis and power this sample size is calculated.

Calculations were not based on a predetermined hypothesis. However, the sample size determination was based on an estimated 95% RPA-LF sensitivity compared to the gold standard and a specificity of 95%. The goal was to evaluate the diagnostic efficacy of a new molecular method compared with standard parasitological and molecular methods.

Sample size was estimated using the formula to estimate a proportion (2), utilizing the parameters:

Confidence Interval 95 % =Z value: 1.96

Error (d^2^): 4% = 0.04

Sensitivity (P): 95% = 0.95

n=	(1.96)^2^ * 0.95 (1- 0.95)

(0.04)^2^

n= 114.05 ~115

Sample size was adjusted by estimating a 5% (0.05) loss of enrolled participants, according with Gupta recommendations (3).

n=	n_0xxxxx_

1-p

n=	115

1-0.05

n=121

We modified the paragraph (lines 148 – 150 on initial version). The new sentence in the final version reads as follow (lines: 150 – 153)

*“Sample size was calculated using the formula to estimate a proportion [22,23]. One hundred fifteen participants were obtained with an expected 95% sensitivity for RPA-LF, 4% error and confidence interval of 95%. It was adjusted by 5% loss of enrollment patients, reaching final sample size of 121 participants [24]”.*

2) Line  253: Are 'the samples' the swabs for qPCR?

Yes, they are swabs as shown on figure1.

# RESULTS

- **Reviewer #1: (No Response)**
- **Reviewer #2:**

1) Line 369/370: the difference in 'results' obtained in the lab versus the field. Details are outlined in Table 3.From these results it is clear that the main differences are TP (72 vs 62) and FN (11 vs 21), of course it is obvious that field analysis will never perform as good as good as a lab context, but the question here is how did these 2 difference occur. By providing the formula used to calculate these values, and indicating which factor contributed to the decrease of TP and increase of FN, the work could become a textbook example of how to go about doing these sort of  things. Again, the quality of the work, the methodology and paper is outstanding, and by adding this info, 'I' would even be tempted to use this paper as a textbook/lecture example in advanced molecular parasitology classes.

The reasons for these differences still need to be determined. The Discussion section of the original submission included some potential reasons and added suggestions for improving the test. First version lines: 433 – 449 and final version lines 434 – 450.

- **Reviewer #3:**

1) Lines 334-336 If the sample size was estimated to be 118, why did the authors include 128 patients?

There was an error in the original manuscript. The final sample size was 121, but we enrolled 128 participants. Communications between the community health workers and primary health facility in Tumaco city was difficult, which resulted in the enrollment of seven additional participants. This issue was reported to the IRB.

Nevertheless, it is important to emphasize that these patients were going to be diagnosed by standard of care methods even in the absence of this study. Additionally, RPA-LF sampling is a non-invasive procedure (placing filter paper on the lesion) and consequently there was no added harm to the patients. For the sake of transparency, we considered important to present the results as they happened in real life.

# CONCLUSIONS

- **Reviewer #1: (No Response)**
- **Reviewer #2:**

1) One last question however, RPA-LFA is not the cheapest technique, and by adding a 'cheap' readout tool in the future, the 'practicality' of the cost could further be an issue. Would it be possible for the authors just to add a few details on the current cost of the RPA-LFA (something that should be similar to RPA-LFAs for other diseases such as trypanosomosis) versus the cost of the 'current' most used golden standard in the field. the latter will be very different depending on the disease studied. It's not crucial for the quality of the paper, but it just allows the reader to think about the bigger picture and out everything in perspective.

The approximate (research setting) cost of RPA-LF is 3-4 USD per test. We estimate that large-scale production should lower the cost to 1-2 USD per test. We plan to address the economical evaluation in a future manuscript.

- **Reviewer #3:**

1) Line 433 - 443    Could sensitivity have been impacted by first doing swab sampling for qPCR and only then filter disks sampling for RPA-LF?

Our reasoning at the beginning of the study was that swabbing of the lesion as the first sampling method would stimulate fluids secretion favoring the absorption of amastigote-laden material with the filter paper (Field scenario). Additionally, utilization of randomization protocols for community health workers in the field would have been logistically burdensome. On the other hand, the sampling order in the reference laboratory scenario was randomized, and resulted in higher sensitivity. However, we do not know whether randomization was the main cause for the higher sensitivity of RPA-LF in the reference laboratory. We expect that in further studies randomization will be considered to identify all the factors influencing the test’s sensitivity.

2) Line 445 - 449    Is the misinterpretation of banding patterns also linked to the <90% specificity of RPA-LF in both field and ref lab?

In general, reading of the lateral flow strips could be misleading when very fine bands appear, giving false positive results. For this reason, it is important to include negative samples in each batch. On the other hand, false negative results are usually the result of lower sensitivity of RPA-LF compared with quantitative PCR.

# EDITORIAL AND DATA PRESENTATION MODIFICATIONS

- **Reviewer #1: (No Response)**
- **Reviewer #2: See other sections**
- **Reviewer #3:**
  1) Line 59 remove the '('
  2) Line 63 duplicated 'in'
  3) Line 161 correct 'performed' to 'perform'
  4) Line 351 duplicated 'in'
  5) Line 397 correct 'Ddiagnotic'

All the edition errors were modified as suggested. Lines in final version are: 60, 64, 163, 352, and 398.

# SUMMARY AND GENERAL COMMENTS

- **Reviewer #1:**

The authors had previously established a RPA-LF protocol for CL diagnosis (Ref 21). I only have minor comments and suggestions below.

1) L.33: in the field?
2) L.63: in both
3) L.225: Leishmania in italic

Modifications were made according to suggestions. Lines in final version are: 34, 64, and 228.

4) L.308-313: The relatively frequent occurrence of contaminations during the RPA protocol, especially in the field, could be explained a bit more in details and discussed in the appropriate section (possible reasons for such contaminations and ways to avoid it in the future).

We add this paragraph to final version: Lines: 452 - 457.

*“Some samples presented contamination. Yet, the areas of highest contamination risk are those found in labs where daily PCR work produce large numbers of amplicons. In the field, sampling with disposable materials offer lower risk of contamination. However, the local clinic should be aware of amplicons and establish separate areas for obtaining samples, extracting DNA and running the molecular test”*

- **Reviewer #2: Very clear paper**.
- **Reviewer #3: (No Response)**

**OTHERS CHANGES**

- We add 2 coauthors to manuscript line 6. Their role in the study were:

Thomas Shelite^4^: evaluated the batches of RPA-LF using Leishmania Viannia spp. available in UTMB, troubleshot any problems related to the test, and coordinated the delivered of reagents to CIDEIM.

Peter Melby^4^ participated in the development of the RPA-LF in UTMB and contribute to the writing of the manuscript.

- We added some minor editions to the final version:

| **Line , first version** | **Line, change in final version** |
| --- | --- |
|  | 265:We add this phrase and a reference:  “and 2.75µL Nuclease-Free Water (32)”. |
| 54: NTDs (Neglected Tropical Diseases) | 55: Neglected Tropical Diseases (NTDs) |
| 81: According to national guidelines | 82: According to Colombian National Guidelines |
| 87: Currently available diagnostic methods | 88: Currently available diagnostic methods for CL |
| 95: referral | 96: reference |
| 245: when amastigotes were visualization | 242 - 243: intra or extracellular amastigotes were observed |
| 310: and 4 in | 313: and 4 from |
| 436: lower than that | 437: lower than |
| 438: by further simplification | 439: by further simplification of the process |
| 485: strengthens | 494: strengths |
| 489: lesion | 498: lesions |
| Table 2  *L.(V) panamensis* 64 (94.8*)*  *L.(V) braziliensis* 5 (7.2) | *L.(V) panamensis* 64 (94.8*)*  *L.(V) braziliensis* 5 (7.2) |
| 364: n=65 94.2 | 365: n=64 94.8 |

- PLOS authors have the option to publish the peer review history of their article

We decided not to publish the peer review history.

- Data requirements: Please note that, as a condition of publication, PLOS' data policy requires that you make available all data used to draw the conclusions outlined in your manuscript. Data must be deposited in an appropriate repository, included within the body of the manuscript, or uploaded as supporting information. This includes all numerical values that were used to generate graphs, histograms etc

All raw data were included as supporting information and will be available as:

S1 Figure 1

S2 Figure 3

S3 Table 2_demographics

S4 Table 2_lesions

S5 Table 3

S6 Table 4

- Figure Files:
  While revising your submission, please upload your figure files to the Preflight Analysis and Conversion Engine (PACE) digital diagnostic tool, [https://pacev2.apexcovantage.com](https://pacev2.apexcovantage.com/). PACE helps ensure that figures meet PLOS requirements.

All figures were approved by PACE digital diagnostic tool. We are attaching figures 1,2 and 3.

- Reproducibility: To enhance the reproducibility of your results, PLOS recommends that you deposit laboratory protocols in protocols.io, where a protocol can be assigned its own identifier (DOI) such that it can be cited independently in the future.

We decided not to publish the laboratory protocols because there are previous publications referenced in our manuscript that describe the corresponding protocols in the Mat. & Methods section.

**References**

1. Gordis L. Epidemiology. Fifth. Ed. 2014. Chapter 5, Assesing the Validity and Reliability of Diagnostic and Screening Tests; pp. 88–115.
2. Hajian-Tilaki K. Sample size estimation in diagnostic test studies of biomedical informatics. J Biomed Inform. 2014;48: 193–204. doi:10.1016/j.jbi.2014.02.013.
3. Gupta KK, Attri JP, Singh A, Kaur H, Kaur G. Basic concepts for sample size calculation: Critical step for any clinical trials! Saudi J Anaesth. 2016;10: 328–331. doi:10.4103/1658-354X.174918.
